# Pharmacological Human Enhancement: An Overview of the Looming Bioethical and Regulatory Challenges

**DOI:** 10.3389/fpsyt.2020.00053

**Published:** 2020-02-17

**Authors:** Giovanna Ricci

**Affiliations:** Section of Forensic Medicine, School of Law, University of Camerino, Camerino, Italy

**Keywords:** cognitive enhancement, human enhancement, smart drugs, nootropics, bioethics

## Abstract

Cognitive enhancement, a rather broad-ranging principle, can be achieved in various ways: healthy eating and consistent physical exercise can lead to long-term improvements in many cognitive domains; commonplace stimulants such as caffeine, on the other hand, temporarily raise levels of alertness, attentiveness, and concentration; sedative substances are also used as an indirect form of enhancement to relax before an exam or an important meeting. Such approaches raise no ethical issue. Nonetheless, clinical research has led to the off-label use of drugs called nootropics or “smart drugs”, which can, under certain conditions, elicit some degree of cognition-improving effects: methylphenidate and modafinil can enhance working memory and concentration in healthy individuals, although the significance and effectiveness of such applications are dubious. Such “cognitive enhancement” methods, however, do raise multiple ethical issues, and their contentious nature has caused bioethical authorities to lay out opinions and recommendations meant to regulate their use. Most notably, the Italian Committee on Bioethics has extensively dealt with the spread of nootropics, which resulted in the Italian Code of Medical Ethics including “cognitive enhancement” drugs and their prescription by doctors as critical points, along with cosmetic surgery (the latest version of the Code, updated in December 2017, deals with the two separately, in Article 76 and 76 BIS). The United States Presidential Commission for the Study of Bioethical Issues broadened the scope of cognitive enhancement techniques so as to include neural modifiers, i.e. mechanisms of brain and nervous system change: a much wider array of interventions, technologies, behaviors, and environmental conditions that may potentially affect several aspects of the human brain and nervous system. The potential of neuroscience to profoundly reshape society is nothing short of mind-blowing.

## Introduction

The ever-growing use of nootropics—also known as “smart drugs”—such as modafinil, piracetam, and methylphenidate by healthy users seeking to enhance their cognitive capabilities has led to widespread social alarm. Although currently-available nootropics offer only modest improvements in terms of cognitive performance, more effective compounds are likely to be developed in the near future, and the off-label use of such substances will probably rise as well. According to recent surveys, the use of such drugs by healthy scholars or by professionals in the ever more competitive labor markets may become commonplace in the short term (1, 2). Methylphenidate, modafinil, amphetamine, and dextroamphetamine are stimulants that inhibit dopamine and norepinephrine reuptake in the brain, affecting cognition and pleasure (3), and have gained popularity as “study drugs”. In 2011 in Germany, Franke et al. reported a prevalence of 1.55% pharmaceutical cognitive enhancer users in a sample of 1,035 pupils from vocational and grammar schools, and a prevalence of 0.78% users in 512 students in medicine, pharmacy, and economics (4). In 2015 in the Swiss canton of Zurich, Liakoni et al. reported a prevalence of 54.5% users among 1,139 students from vocational schools and upper secondary schools (9.2% of prescription drug users, 44% of which used methylphenidate) (5). The same year in the Netherlands, Schelle et al. reported a prevalence of 1.7% prescription drug users for cognitive enhancement in a sample of 1,572 university students (methylphenidate or beta-blockers) (6). In 2016 in Germany, Dietz et al. reported a prevalence of 19% prescription and illicit drug users for cognitive enhancement among 1,021 people working in the field of economics; methylphenidate, amphetamine, and modafinil were the most commonly used prescription drugs (7).

The use of cognitive enhancement has raised ethical concerns. Additionally, while their effects on cognitive enhancement is arguable, the use of methylphenidate, modafinil, amphetamine, and other prescription drugs involves health risks, including dependence, tolerance, and cardiovascular, neurologic, and psychological disorders (8). To deal with these ethical and health issues, international laws are adapting. The author aimed to provide an overview of the situation regarding the ethical and regulatory implications of nootropic use by focusing on the Italian Code of Medical Ethics and drawing a comparison with the international regulations.

## Cognitive Enhancement: The Italian Code of Medical Ethics and the Italian Committee for Bioethics Offer Valuable Perspectives

In 2014, the article 76 of the Italian Code of Medical Ethics first attempted to define human enhancement and medical practices and treatments associated (9, 10). Article 76 was meant to clarify the relation between this new field of medical practice and professional ethics. Indeed, the inclusion of medical/pharmacological enhancement practices in the Italian Code allowed medical treatments going beyond conventional therapeutic goals, as long as ethical and clinical criteria are met. Still, the problematic application of those criteria to treatments aiming at human enhancement generated various issues warranting an in-depth reflection. The indisputable importance of the newly-available and ever-evolving human enhancement techniques is addressed in the new version of the article of the Italian Code of Medical Ethics [articles 76 and 76 bis (11), released by the National Federation of Physicians’ and Dentists’ Orders on the 15^th^ of December 2017] which further clarified the ethical and professional standards that health care professionals should meet when evaluating pharmacological enhancement through nootropics. Cognitive/physical enhancement and aesthetic medicine are addressed separately in the new version of the article (articles 76 and 76 bis, respectively). The new article 76 states that medical doctors being asked to provide or prescribe non-therapeutic treatments aiming at achieving cognitive/physical enhancement should always be guided by the highest standards of respect and protection for human dignity, identity, and integrity, and operate in accordance with the principles of proportionality and precaution.

Information also has become essential: medical doctors are required to obtain written informed consent, after explaining all possible risks arising from the proposed treatment, and should turn down any request for treatment or prescription that they consider disproportionate or unacceptably risky due to their invasive or irreversible nature. In order to fully understand the evolution of the concept of health, it is necessary to explore the changes that have occurred over time, taking into account several Italian constitutional precepts and opinions by the Italian Bioethics Committee. The new concept of the right to enjoy good health has apparently been broadened to include the right to become “better”, i.e. more performant or better-looking. A new meaning of “health” was introduced, unrelated to the concept of “care” and inspired by a peculiar concept of “happiness”, a principle not found in the Italian Constitution. The relation between this new field of medical practice and professional ethics is quite complex. Article 76 of the Italian Code of Medical Ethics allows medical treatments beyond the usual therapeutic goals. However, extreme caution is needed due to uncertain and insufficient evidence regarding the safety of most nootropics: in a 2013 opinion, the Italian Committee for Bioethics argued that more comprehensive research is necessary to outline the benefits and risks of nootropics, upon which experts did not reach consensus. In the meantime, more reliable means to enhance cognitive functions should be promoted and prioritized: education, constant intellectual exercise and learning, a rewarding social life and interactions, and a stimulating and healthy lifestyle. As discussed by the Committee, such an approach is certainly more demanding and time-consuming than taking a supposedly “enhancing” drug, but it is much better in terms of development of personal identity, establishment of satisfactory interrelationships, self-esteem, and self-fulfillment (12). Such remarks and concerns seem to have been specifically addressed in the updated article 76 of the Italian Code of Medical Ethics, and most notably by the United States Presidential Commission for the Study of Bioethical Issues, among others.

### As Nootropics use Becomes Rife, Ethical Doubts Linger

In April 2015, in the United States (13), a controversy arose around the growing use of Adderall®, a combination of amphetamines, in young adults without therapeutic purpose. Amphetamines and other stimulants are used off label by individuals seeking to heighten their competitive advantage by working longer and with a greater degree of attentiveness while sleeping less (14). Similarly, an ‘epidemic’ of amphetamine use by students seeking top grades and better test scores (15) was reported. These psychoactive drugs may alter the perception of reality of users, including their own actions and subsequent consequences (16). Such cognitive enhancement methods are still being explored: it is unclear whether enhancers are actually effective or have the potential to alter and manipulate memories, moral autonomy, and personality. It may arguably be appropriate to draw distinctions among different tools and methods to achieve enhancement, based on their risk-benefit ratios. It is worth noting that certain techniques are more risky, and others are more likely to succeed. For instance, pharmaceutical enhancers are currently considered the method most likely to succeed in achieving a reasonable level of cognitive improvement, in safe enough a manner (17). Greater risk is entailed by brain manipulation and stimulation; nonetheless, it is not clear yet whether those risks should be considered so high that competent individuals are not allowed to consent to them in light of different risks that individuals are legally allowed to take [for cosmetic improvements for example (18)]. The fundamental question is: do enhancers conflict with societal ethical values? According to some, performance-enhancing drugs should be banned, since they might create unfair competition. Moreover, mainstream use might indirectly pressure non-users to take nootropics, in the attempt to remain competitive. Is it conceivable, however, to restrict the freedom and autonomy of everyone out of fear that it may influence someone else’s choices? The use of nootropics is often viewed as unjustifiable; yet, libertarian approaches tend to advocate for the individual right to determine whether such risks are worth taking (19).

## What Ethical Boundaries Should be Outlined?

Where should an “ethical line” be drawn, if at all, between merely treating or preventing disorders and deficiencies in order to achieve ‘normal’ functioning, and resorting to drugs or devices for improvement or “enhancement”, maybe to the point of becoming ‘super-human’? Should resources be spent trying to turn average people into smarter and/or better performing versions of themselves? Even though the use of nootropics for the purpose of cognitive enhancement has been getting more and more widespread over the past years and such drugs are broadly perceived to somehow improve academic and professional performances, not enough empirical evidence supports the assumption that these drugs indeed give rise to substantial enhancement in healthy users. In that respect, a 2017 survey of 898 undergraduates, who were not diagnosed with attention deficit hyperactivity disorder (ADHD), reported that the use of cognitive enhancers did not result in an increase in the grade point average or an advantage of any sort over non-users (20). That is further confirmation that research on nootropics still appears to be inconclusive in terms of clarifying and defining how such drugs act as mind stimulants (21). Remarks from the Australian Alcohol and Drug Foundation (22) have cast doubt on the actual cognitive benefits of most nootropics, underlining that scientific studies showed only little to no benefits for cognitive enhancement in healthy individuals, while the associated side effects do pose health risks (23). Furthermore, granted that the use of nootropics may somehow help to bear fatigue, boredom, or procrastination, there is no evidence suggesting that they can actually make people smarter. Besides, their effects are apparently temporary, lasting until their metabolization and elimination (24). Some of these drugs can be addictive and have a range of side effects particularly harmful to young people, as their brains continue to develop into their mid-twenties. Medical associations and institutions should urgently devise clear guidelines in order to help medical doctors and health care institutions to face the issue of cognitive enhancement in healthy individuals.

Substances interacting with the mediators of memory and learning circuits such as glutamate, dopamine, and norepinephrine can potentially improve brain function in healthy users, even to the point of improving their baseline levels of functioning. That is already happening: non-medical use of prescription stimulants such as methylphenidate and the illicit use of psychostimulants for cognitive enhancement have risen among adolescents and young adults in schools and college campuses (8, 25, 26). However, there may be health and ethical costs. For instance, an alteration of the glutamatergic system caused by the intake of psychostimulants may impair behavioral flexibility, leading to the development and/or potentiation of addictive behaviors. Methylphenidate may lower drug abuse liability in ADHD patients, but it may also lead to similar behavioral rigidity and increase the risk for addictive or obsessive-compulsive behaviors by affecting glutamatergic signaling (27, 28). Another example of nootropic that has been under great scrutiny in the scientific community is modafinil, a compound structurally similar to methylphenidate and currently approved by the US Food and Drug Administration (FDA) for the treatment of narcolepsy, obstructive sleep apnea, excessive daytime sleepiness in adults and children (29), and shift-work disorder. The effects of modafinil on alertness and wakefulness in healthy individuals who are not sleep-deprived, and its military applications (30), has led to its use as a cognitive enhancer. Still, it has been observed that modafinil at certain doses can cause a reduction in *N*-methyl-d-aspartate (NMDA) receptor levels, impairments in short-term and long-term brain plasticity, which was also observed with methylphenidate (31). With such a record, still under-documented and indicating potential health hazards, it is of paramount importance to be able to rely on unequivocal standards, recommendations, and guidelines from scientific institutions in order to regulate and govern the growing diffusion of nootropics. Most academic institutions, companies, and business associations have not taken a definite stance yet, either in favor or opposed to their use; such ambiguity leaves the issue of cognitive enhancement in a sort of a limbo: it is criticized when openly addressed, yet the cultural environments of business and academia, which are highly competitive settings, somehow support the use of these drugs in private. Two main arguments are frequently expressed by ethicists opposed to cognitive enhancement: firstly, it runs counter to the absolute value of authenticity and secondly, it is tantamount to a form of unfairness and cheating. Still, in the view of the author, both those arguments fail to thoroughly account for the individual and social factors that may get people to use or oppose the use of nootropics. The intuition that the use of cognitive enhancement by healthy people is unfair can be explained both philosophically and psychologically (32). As mentioned above, the ethical and philosophical ramifications of cognitive enhancers are somewhat complex, and lend themselves to multiple reflections and interpretations.

## US Presidential Commission for the Study of Bioethical Issues: Neural Modification has the Potential to Reshape Society

In 2015, the US Presidential Commission for the Study of Bioethical Issues (Bioethics Commission) released a Report (33) on the issue of cognitive enhancement, and laid out its findings and recommendations for the scientific community. The Committee widened the scope of the debate by including all forms of neural modification. The relevance of future research developments on the matter is nothing less than mind-blowing: it has the potential to profoundly reshape society in the years to come. “Gray Matters” (34) attempted to answer some of those thorny questions, and outline a tenable path for informed, sensible, and productive exchanges in order to start a public discussion centered on cognitive enhancement. A related issue is the current medical acceptance, or even endorsement, of interventions intended to restore or sustain “normality.” Such a stance apparently adheres to the idea of a set of socio-cultural requirements to function “normally”, considering abnormal or anti-social any deviation from established standards. What posture should be taken up when some people strive for a level of “optimal functioning”, seeking what they personally view as the nadir of the good life and what about society setting requirements that individuals in special positions and professions (e.g. police officers, doctors, pilots, or military personnel) are supposed to meet so as to achieve an “acceptable” level of optimal functioning? Medicine’s essential, commendable function in service of achieving a good, healthy life is not to be considered as automatically extendable to living a “great” life, or to attaining above average, excellent performances in a socially-sanctioned function or service (34). Hence, justifications for specialized enhancements meant to achieve extraordinary lifestyles or performances will not necessarily be obtained starting from and according to medical principles (35). Furthermore, as we argued before, cognitive enhancement is not the end of the line, far from it: unremitting scientific progress and advancements make it essential to move beyond it and toward a broader array of interventions, technologies, behaviors and environmental conditions that can affect many aspects of the human brain and nervous system. The expression ‘neural modifiers’ is frequently used to refer to this wider pool of brain mechanisms and nervous system variations. Three general categories of neural modifiers can be identified: those meant to keep, or even strengthen, neural health but only within its normal boundaries; those for the purpose of treating an illness or a disorder; and lastly, the most controversial type: those conceived to improve or enhance one’s capabilities beyond their normal, average function (36, 37). No category of neural modifiers—even those that make us better than ‘normal’—is in the author’s estimation inherently ethical or unethical. Instead, each form of neural modifier should be assessed on its own terms and merits, on a case-by-case basis, so as to best determine whether its use is ethical in a given context. Stakeholders and members of the public need to ask questions to make this ethical assessment, such as: what is the method and ultimate goal of the neural modifier? Is it safe and effective for that purpose? Who is choosing the modifier, and is that choice free of coercion and pressure? And the list goes on.

Neuroscience research holds tremendous promise, but it is also the subject of excessive media hyperbole. It is also worth noting that neurotechniques have wide-ranging applications in the legal realm as well: namely, the identification of biomarkers for anxiety-related conditions such as post-traumatic stress disorder, which may result in better therapeutic options and more accurate evaluations of psychological disorders; the implications in tort as well as in criminal law, e.g. for the calculation of compensatory damages, will be remarkable (38, 39). Still, the Bioethics Commission cautions against unrealistic claims and exaggeration. Conversations about neural modification, and cognitive enhancement in particular, generate hype among scholars, journalists, and the public. For example, the potentially enhancing effects of drugs such as methylphenidate and amphetamines, which are normally used to treat patients suffering from ADHD have often been overstated by the media (40). Furthermore, according to the Commission, existing, low-tech strategies ought to be prioritized by scholars in terms of new research and its funding, rather than highly technological methodologies and neural modifiers, which are frequently costly, and whose benefits many believe to be dubious or minimal (41, 42). Moreover, research aimed at treating neurological disorders, promoting mental health, and ending suffering should be at the forefront. The committee also advocates for research on the prevalence, possible benefits, and risks of new neural modifiers for the development of neural functions, and stresses equality of opportunities as an added value: access to neural enhancers that are proven to be beneficial, effective, harmless, and morally acceptable should be ensured for anyone (43, 44). Nonetheless, in light of the innovative nature of those tools, solid guidance about the use of neural modifiers needs to be provided for all those involved: patients, parents, employers, specialists, and educators; the potential risks and benefits must be further researched and disclosed. Neurologically sound minors and adolescents should not be prescribed drugs with uncertain or unverified benefits. Such decisions have in fact a higher degree of ethical complexity when they affect children, since minors lack the legal and ethical capacity to consent to treatment and are more vulnerable to external influences and even coercion (45).

## Conclusions

It is safe to assume that the lack of clear guidance for physicians has been causing inequality in patient access. Some healthy patients have been legitimately prescribed nootropics by doctors in favor of cognitive enhancers; is it not unfair that others have been breaking the law in order to gain access to the same drugs? It is the medical community’s duty to evaluate the risk-benefit ratio of neural modifiers for cognitive enhancement in the healthy. More research is needed to figure out the possible hazards entailed by the use of nootropics. If a thorough review of the evidence confirms that a certain medicine has a benefit/harm profile that warrants its use by healthy patients, appropriate steps should be taken by the medical community in order to ensure that patients have been informed of their options. Conversely, if the potential harms of cognitive enhancement for the healthy are deemed to be too great, doctors should strive to educate their patients and the public at large as to the potential hazards of nootropic use. According to Roache and Savulescu, nobody can know for sure how to use these drugs safely and effectively (46); in fact, there is a dearth of scientific evidence as to the large-scale use of nootropics [or other techniques such as transcranial electrical stimulation (47)] aimed at human enhancement. As pointed out by the British Medical Association in 2015, it is difficult to improve the cognitive capabilities of healthy individuals already functioning at an optimum level (48). Currently available evidence suggests that healthy individuals seeking to preserve or enhance their cognitive capabilities should avoid pharmacological cognitive enhancers and focus instead on a healthy and rewarding lifestyle; that is in our opinion a sensible and well-balanced approach. More thorough research is undeniably necessary, if we are to ever dispel the lingering doubts about cognitive enhancement: medical professional organizations will then be able to outline evidence-based professional criteria, recommendations, and guidelines for large-scale cognitive enhancement for healthy users. Ultimately, in the view of the author, the fundamental standard to be met is safety: given the scarcity of scientific evidence as to the actual enhancing capabilities of nootropics and their potential for unwanted harmful side-effects, recommendations from international health care bodies should be quite strict and essentially against their use. After all, there are several instances of protective/restrictive measures and approaches that are dictated by the prioritization of safety, when it comes to techniques and drugs aimed at improving performance rather than treating diseases: the use of performance-enhancing drugs in sports certainly falls within that category. Erythropoietin abuse among cyclists has been tackled and harshly punished in the name of safety for athletes, and an approach along those lines, the author believe, could also be adopted toward nootropics use in society at large.

## Author Contributions

The author designed, drafted, and wrote the review paper.

## Conflict of Interest

The author declares that the research was conducted in the absence of any commercial or financial relationships that could be construed as a potential conflict of interest.

## References

[1] Abelman DD Mitigating risks of students use of study drugs through understanding motivations for use and applying harm reduction theory: a literature review. Harm Reduct J (2017) 14:68. 10.1186/s12954-017-0194-6 28985738 PMC5639593

[2] Zaami S Varì MR Tini A Marinelli E Cognitive enhancing drugs: a future challenge for the workplace? Eur Rev Med Pharmacol Sci (2019) 23(12):5027–9. 10.26355/eurrev_201906_18165 31298356

[3] Smith ME Farah MJ Are prescription stimulants “smart pills”? The epidemiology and cognitive neuroscience of prescription stimulant use by normal healthy individuals. Psychol Bull (2011) 137:717–41. 10.1037/a0023825 PMC3591814 21859174

[4] Franke AG Bonertz C Christmann M Huss M Fellgiebel A Hildt E Non-medical use of prescription stimulants and illicit use of stimulants for cognitive enhancement in pupils and students in Germany. Pharmacopsychiatry (2011) 44:60–6. 10.1055/s-0030-1268417 21161883

[5] Liakoni E Schaub MP Maier LJ Glauser GV Liechti ME The use of prescription drugs, recreational drugs, and “soft enhancers” for cognitive enhancement among Swiss secondary school students. PloS One (2015) 10:e0141289. 10.1371/journal.pone.0141289 26505633 PMC4624689

[6] Schelle KJ Olthof BM Reintjes W Bundt C Gusman-Vermeer J van Mil AC A survey of substance use for cognitive enhancement by university students in the Netherlands. Front Syst Neurosci (2015) 9:10. 10.3389/fnsys.2015.00010 25741248 PMC4330699

[7] Dietz P Soyka M Franke AG Pharmacological neuroenhancement in the field of economics – Poll results from an online survey. Front Psychol (2016) 7:520. 10.3389/fpsyg.2016.00520 27148128 PMC4835716

[8] Carlier J Giorgetti R Varì MR Pirani F Ricci G Busardò FP Use of cognitive enhancers: methylphenidate and analogs. Eur Rev Med Pharmacol Sci (2019) 23:3–15. 10.26355/eurrev_201901_16741 30657540

[9] Montanari Vergallo G Busardò FP Zaami S Marinelli E The static evolution of the new Italian code of medical ethics. Eur Rev Med Pharmacol Sci (2016) 20:575–80. 26914136

[10] Ricci S di Luca A di Luca NM Comment on “the static evolution of the new Italian code of medical ethics”. Eur Rev Med Pharmacol Sci (2016) 20:2753–4. 27424967

[11] Federazione Nazionale degli Ordini dei Medici Chirurghi e degli Odontoiatri (2014). Codice di Deontologia Medica. Article 76 was modified on December 17, 2017.

[12] Italian Committee for Bioethics (2013). Neuroscienze e Potenziamento Cognitivo Farmacologico: Profili Bioetici. Available online at: http://bioetica.governo.it/media/3485/p106_2013_enhancement-cognitivo_it.pdf (Accessed December 20, 2019).

[13] Schwarz A (2015). Workers seeking productivity in a pill are abusing ADHD drugs. New York Times. Available online at: https://www.nytimes.com/2015/04/19/us/workers-seeking-productivity-in-a-pill-are-abusing-adhd-drugs.html (Accessed December 20, 2019).

[14] Baratgin J Douven I Evans JSBT Oaksford M Over D Politzer G The new paradigm and mental models. Trends Cogn Sci (2015) 19:547–8. 10.1016/j.tics.2015.06.013 26412091

[15] Ford JA Pomykacz C Non-medical use of prescription stimulants: a comparison of college students and their same-age peers who do not attend college. J Psychoact Drugs (2016) 48:253–60. 10.1080/02791072.2016.1213471 27541987

[16] Lavazza A Erasing traumatic memories: when context and social interests can outweigh personal autonomy. Philos Ethics Humanit Med (2015) 10:3. 10.1186/s13010-014-0021-6 25879841 PMC4340636

[17] Colucci L Bosco M Rosario Ziello A Rea R Amenta F Fasanaro AM Effectiveness of nootropic drugs with cholinergic activity in treatment of cognitive deficit: a review. J Exp Pharmacol (2012) 4:163–72. 10.2147/JEP.S35326 PMC4863555 27186129

[18] British Medical Association Boosting your brainpower: ethical aspects of cognitive enhancement, discussion paper. London (UK): British Medical Association (2007). Available online at: https://pdfs.semanticscholar.org/08b0/bcfadb2f4b07823195f047e03184e4f7992a.pdf (Accessed December 20, 2019).

[19] Cakic V Smart drugs for cognitive enhancement: ethical and pragmatic considerations in the era of cosmetic neurology. J Med Ethics (2009) 35:611–5. 10.1136/jme.2009.030882 19793941

[20] Arria AM Do college students improve their grades by using prescription stimulants nonmedically? Addict Behav (2017) 65:245–9. 10.1016/j.addbeh.2016.07.016 PMC5140739 27469455

[21] Urban KR Gao WJ Performance enhancement at the cost of potential brain plasticity: neural ramifications of nootropic drugs in the healthy developing brain. Front Syst Neurosci (2014) 8:38. 10.3389/fnsys.2014.00038 24860437 PMC4026746

[22] Alcohol and Drug Foundation (2019). Nootropics. https://adf.org.au/drug-facts/cognitive-enhancers Last updated: May 28th.

[23] Hall WD Lucke JC The enhancement use of neuropharmaceuticals: more scepticism and caution needed. Addiction (2010) 105:2041–3. 10.1111/j.1360-0443.2010.03211.x 21054609

[24] De Jongh R Bolt I Schermer M Olivier B Botox for the brain: enhancement of cognition, mood and pro-social behavior and blunting of unwanted memories. Neurosci Biobehav Rev (2008) 32:760–76. 10.1016/j.neubiorev.2007.12.001 18295885

[25] Busardò FP Kyriakou C Cipolloni L Zaami S Frati P From clinical application to cognitive enhancement: the example of methylphenidate. Curr Neuropharmacol (2016) 14:17–27. 10.2174/1570159X13666150407225902 26813119 PMC4787280

[26] Madan CR Augmented memory: a survey of the approaches to remembering more. Front Syst Neurosci (2014) 8:30. 10.3389/fnsys.2014.00030 24624063 PMC3939671

[27] Frati P Kyriakou C Del Rio A Marinelli E Vergallo GM Zaami S Smart drugs and synthetic androgens for cognitive and physical enhancement: revolving doors of cosmetic neurology. Curr Neuropharmacol (2015) 13:5–11. 10.2174/1570159X13666141210221750 26074739 PMC4462043

[28] Newman LA McGaughy J Adolescent rats show cognitive rigidity in a test of attentional set shifting. Dev Psychobiol (2011) 53:391–401. 10.1002/dev.20537 21365641 PMC10722888

[29] Sullivan SS Current treatment of selected pediatric sleep disorders. Neurotherapeutics (2012) 9:791–800. 10.1007/s13311-012-0149-2 23055049 PMC3480565

[30] Taylor GP Keys RE Modafinil and Management of Aircrew Fatigue. Washington, DC, 2003: United States Department of the Air Force (2003).

[31] Urban KR Li YC Gao WJ Treatment with a clinically-relevant dose of methylphenidate alters NMDA receptor composition and synaptic plasticity in the juvenile rat prefrontal cortex. Neurobiol Learn Mem (2013) 101:65–74. 10.1016/j.nlm.2013.01.004 23333502 PMC3602399

[32] Bedzow I The confused ethics of cognitive enhancers. J Clin Psychiatry Neurosci (2018) 1:12–4.

[33] Presidential Commission for the Study of Bioethical Issues Gray Matters: Topics at the Intersection of Neuroscience, Ethics, and Society. In: Presidential Commission for the Study of Bioethical Issues (2015). Available online at: https://bioethicsarchive.georgetown.edu/pcsbi/node/4704.html (accessed December 20, 2019).

[34] Hofmann B Limits to human enhancement: nature, disease, therapy or betterment? BMC Med Ethics (2017) 18:56. 10.1186/s12910-017-0215-8 29017486 PMC5635529

[35] Shook JR Galvagni L Giordano J Cognitive enhancement kept within contexts: neuroethics and informed public policy. Front Syst Neurosci (2014) 8:228. 10.3389/fnsys.2014.00228 25538573 PMC4256981

[36] Allen A Strand NK Cognitive enhancement and beyond: defining the scope. Trends Cogn Sci (2015) 19:549–51. 10.1016/j.tics.2015.08.001 26412093

[37] Dresler M Sandberg A Bublitz C Ohla K Trenado C Mroczko-Wasowicz A Hacking the brain: dimensions of cognitive enhancement. ACS Chem Neurosci (2019) 10:1137–48. 10.1021/acschemneuro.8b00571 PMC6429408 30550256

[38] Freedman D Zaami S Neuroscience and mental state issues in forensic assessment. Int J Law Psychiatry (2019) 65:101437. 10.1016/j.ijlp.2019.03.006 30952490

[39] Marinelli S Neuroscience and law: revolutionizing criminal proceedings, despite a few pitfalls. Eur Rev Med Pharmacol Sci (2019) 23:6005–7. 10.26355/eurrev_201907_18408 31364101

[40] Partridge BJ Bell SK Lucke JC Yeates S Hall WD Smart drugs “as common as coffee”: media hype about neuroenhancement. PloS One (2011) 6:e28416. 10.1371/journal.pone.0028416 22140584 PMC3227668

[41] Ilieva I Boland J Farah MJ Objective and subjective cognitive enhancing effects of mixed amphetamine salts in healthy people. Neuropharmacology (2013) 64:496–505. 10.1016/j.neuropharm.2012.07.021 22884611

[42] Ragan CI Bard I Singh I Independent scientific committee on drugs. what should we do about student use of cognitive enhancers? An analysis of current evidence. Neuropharmacology (2013) 64:588–95. 10.1016/j.neuropharm.2012.06.016 22732441

[43] Buchanan A Beyond Humanity: The Ethics of Biomedical Enhancement. New York, NY: Oxford University Press (2011).

[44] Greely H Sahakian B Harris J Kessler RC Gazzaniga M Campbell P Towards responsible use of cognitive-enhancing drugs by the healthy. Nature (2008) 456:702–5. 10.1038/456702a 19060880

[45] Urban KR Gao WJ Psychostimulants as cognitive enhancers in adolescents: more risk than reward? Front Public Health (2017) 5:260. 10.3389/fpubh.2017.00260 29034227 PMC5626934

[46] Roache R Savulescu J Enhancing Conservatism. In: Clarke S Savulescu J Coady T Giubilini A Sanyal S, editors. The Ethics of Human Enhancement: Understanding the Debate. Oxford (UK): Oxford University Press (2016). Chapter 10. 27997092

[47] Lavazza A Transcranial electrical stimulation for human enhancement and the risk of inequality: prohibition or compensation? Bioethics (2019) 33:122–31. 10.1111/bioe.12504 30157289

[48] British Medical Association Cognitive enhancing drugs and the workplace. (2015). Available online at: https://www.bma.org.uk/advice/employment/occupational-health/cognitive-enhancing-drugs (Accessed December 20, 2019).

